# Harnessing big data for health equity through a comprehensive public database and data collection framework

**DOI:** 10.1038/s41746-023-00844-5

**Published:** 2023-05-20

**Authors:** Cameron Sabet, Alessandro Hammond, Nim Ravid, Michelle Sun Tong, Fatima Cody Stanford

**Affiliations:** 1 Georgetown University School of Medicine, Washington, DC USA; 2 Harvard University, Cambridge, MA USA; 3 Division of Hematology/Oncology and Department of Pediatric Oncology, Boston Children’s Hospital, Boston, MA USA; 4 University of California San Francisco School of Medicine, San Francisco, CA USA; 5 Massachusetts General Hospital, Harvard Medical School, Boston, MA USA

**Keywords:** Policy, Funding

## Abstract

The United States Department of Health and Human Services (HHS) pledged $90 million to help reduce health disparities with data-driven solutions. The funds are being distributed to 1400 community health centers, serving over 30 million Americans. Given these developments, our piece examines the reasons behind the delayed adoption of big data for healthcare equity, recent efforts embracing big data tools, and methods to maximize potential without overburdening physicians. We additionally propose a public database for anonymized patient data, introducing diverse metrics and equitable data collection strategies, providing valuable insights for policymakers and health systems to better serve communities.

## Main

In April 2022, Health and Human Services (HHS) pledged 90 million USD to the American Rescue Plan Uniform Data System Patient-Level Submission (ARP-UDS+) funding award in support of new data-driven efforts for Health Resources and Services Administration (HRSA) Health Center Programs to identify and reduce health disparities^1^. This sum is planned to be distributed to 1400 community health centers (CHCs) serving over 30 million Americans^1^. Specifically, this pledge will allow health centers to expand analytics and reporting capabilities to enhance healthcare services while supporting patient-level UDS+ data submissions to collect more precise data on health disparities^2^. We herein propose a national public database featuring voluntarily self-disclosed and deidentified patient data to increase the granularity of health disparities metrics, and propose how this project can incorporate diverse metrics and equitable data collection procedures.

Big data has already been proposed to promote health equity at the federal level. For instance, in 2021, the Agency for Healthcare Research and Quality (AHRQ) released the National Healthcare Quality and Disparities Report (NHQR)^3^. The NHQR is a comprehensive review of American healthcare comparing health outcomes across both state and national levels^4^. This report revealed that, although communities of color have enjoyed increased healthcare access since 2000, racial inequity persisted in 2021 due to lack of focused interventions addressing disparities^3^. While CHCs disproportionately serve marginalized populations, their lack of granular data collection is likely leading to underestimations of health disparities in areas requiring the greatest resource support^4^.

Big data has impacted patient care for decades by helping health insurance companies incentivize preventative care among patients and physicians, ultimately decreasing the use of costly acute care and improving care equity^4^. For instance, the Community Health Needs Assessment (CHNA) in the Patient Protection and Affordable Care Act (ACA) requires CHCs to investigate the biggest health-related challenges in their communities and outline solutions^5^. However, CHNAs often lack subjective data like medical trust and are only collected periodically, producing an unnecessary lag time between when systemic health issues arise and when healthcare providers can identify and respond to them.

Given these challenges with current healthcare metrics, the Centers for Medicare and Medicaid Services (CMS) should develop a national database like the CDC’s Vaccine Adverse Event Reporting System (VAERS), which collects self-reported issues with vaccine reactions, to regularly collect self-reported data on symptoms, diseases, adverse reactions, medical trust, and perceptions of health equity through metrics like transportation time or food insecurity^4^. This platform can also be used to monitor medications while empowering CHCs to pinpoint and respond to systemic issues more quickly. Although the database may be skewed towards higher-income individuals with more time to self-report, the resource can help compare assess healthcare quality without overburdening physicians, ultimately improving equity interventions.

Although the data would be self-reported, the requested data should include many new metrics. For instance, the National Committee for Quality Assurance (NCQA) Healthcare Effectiveness Data and Information Set (HEDIS), the basis for many equity measurement systems, HEDIS was inaugurated in 1991 and continued for 31 years without race or ethnicity included in its nearly one hundred performance measures^6^. These metrics were only introduced to eight of these measurements, and that only in 2022^6^. HEDIS also does not stratify metrics by other determinants of health like food insecurity or employment status^6^. Metrics for further development could also include language access, transportation barriers, and health literacy.

Healthcare organizations can better understand language barriers by offering patient surveys after each visit. Depending on the extent of the issue, Community Health Centers (CHCs) can then adopt interpreter services and track its effectiveness through surveys. To address transportation barriers, patients can opt to disclose transportation service use and reasons for missed appointments. CHCs can then partner with transportation providers like Uber WAV (Wheelchair Accessible Vehicles) and Uber Health to improve patient accessibility and cover for deficiencies in available wheelchair-accessible vehicles^7^. CHCs can also assess the readability of patient education materials and track the effectiveness of health literacy interventions like counseling, visual aids, staff training on health literacy communication, and simplified language in educational pamphlets through validated health literacy screening tools like REALM-SF (Rapid Estimate of Adult Literacy in Medicine - Short Form)^8^. REALM-SF examines abilities to read aloud medical terminology and is available free for download on the National Center for Education Statistics (NCES) website^9^.

To ensure that the system captures data from marginalized populations, we propose CHCs invest in technology infrastructure and staff training to prepare for more comprehensive data collection by, for instance, leveraging telehealth services to provide care management while streamlining data collection through remote patient monitoring and automated post-checkup surveys. Then, outside partnerships with research organizations can help CHCs analyze this data and devise tailored solutions through partnerships with relevant nonprofits like the National Partnership for Women and Families, which has developed best practices for patient engagement in healthcare, and Leapfrog, which helps analyze data to promote healthcare safety and quality^10,11^.

Furthermore, to promote interoperability with electronic health records, we suggest following the technical standards developed by organizations such as the Office of the National Coordinator for Health Information Technology (ONC) and the Health Level Seven International (HL7) organization. These standards ensure that data can be exchanged seamlessly between different healthcare systems while safeguarding self-reported data. We also recommend consulting with the ONC’s Trusted Exchange Framework and Common Agreement (TEFCA) to help establish policies for securely exchanging health information across organizations.

However, marginalized groups must be involved in the design of this database. They could provide input on how to create a user-friendly interface with icons instead of words on buttons to accommodate those with low literacy or limited technological proficiency. The database should also collect data on the widest possible range of demographic factors; provide technical support through online tutorials or help desks staffed by technical support personnel; establish robust privacy policies like strict data access controls, encryption protocols, and other technical safeguards; and implement data quality controls, like automated data validation checks; and seamlessly deliver the data to researchers and policymakers through data dashboards.

As big data revolutionizes the world, we must not forget its potential impact on improving health outcomes and disparities. By investing funds into health centers and hospitals for patient-driven reporting and data collection on race and ethnicity and developing minimum standards for health equity metrics collection and reporting across states, public discourse and media then draw on subsequent patient data analyses to bring greater attention to regional and national healthcare inequities, leading lawmakers to prioritize health equity initiatives and healthcare systems to have a clearer picture of the communities they serve.

### Reporting summary

Further information on research design is available in the Nature Research Reporting Summary linked to this article.

## Supplementary information

Reporting Summary
